# Dietary Chitosan Nanoparticles: Potential Role in Modulation of Rainbow Trout (*Oncorhynchus mykiss*) Antibacterial Defense and Intestinal Immunity against Enteric Redmouth Disease

**DOI:** 10.3390/md19020072

**Published:** 2021-01-29

**Authors:** Fatma Ahmed, Faiza M. Soliman, Mohamed A. Adly, Hamdy A. M. Soliman, Mansour El-Matbouli, Mona Saleh

**Affiliations:** 1 Clinical Division of Fish Medicine, University of Veterinary Medicine, Veterinärplatz 1, 1210 Vienna, Austria; Fatma.Ahmed@vetmeduni.ac.at (F.A.); Mansour.El-Matbouli@vetmeduni.ac.at (M.E.-M.); 2 Department of Zoology, Faculty of Science, Sohag University, Sohag 82524, Egypt; faiza.soliman2019@gmail.com (F.M.S.); Mohamed_adly@science.sohag.edu.eg (M.A.A.); Hamdy_soliman@science.sohag.edu.eg (H.A.M.S.)

**Keywords:** antibacterial defense, chitosan nanoparticles, ERM, intestinal immunity, prophylactic-regimen, rainbow trout, serum, skin mucus, therapeutic-regimen, *Yersinia ruckeri*

## Abstract

Bio-nanotechnology employing bio-sourced nanomaterial is an emerging avenue serving the field of fish medicine. Marine-sourced chitosan nanoparticles (CSNPs) is a well-known antimicrobial and immunomodulatory reagent with low or no harm side effects on fish or their human consumers. In this study, in vitro skin mucus and serum antibacterial activity assays along with intestinal histology, histochemical, and gene expression analyses were performed to evaluate the impact of dietary CSNPs (5 g kg^−1^ dry feed) on rainbow trout resistance against ‘enteric redmouth’ disease. Two treatment conditions were included; short-term prophylactic-regimen for 21 days before the bacterial challenge, and long-term therapeutic-regimen for 21 days before the challenge and extended for 28 days after the challenge. Our results revealed higher antibacterial defense ability and positive intestinal histochemical and molecular traits of rainbow trout after dietary CSNPs. The prophylactic-regimen improved trout health while the therapeutic regimen improved their disease resistance and lowered their morbidity. Therefore, it is anticipated that CSNPs is an effective antibacterial and immunomodulatory fish feed supplement against the infectious threats. However, the CSNPs seem to be more effective in the therapeutic application rather than being used for short-term prophylactic applications.

## 1. Introduction

Yersiniosis or the enteric redmouth disease (ERM) is a systemic bacterial disease of salmonids, mainly in farmed rainbow trout (*Oncorhynchus mykiss*). It is caused by the Gram-negative bacterium *Yersinia ruckeri* and leads to cumulative mortality and high economic losses [1,2]. It is an acute to chronic infectious disease accompanied by systemic hemorrhages [3] along with serious histopathological changes in gills, kidneys, and spleen [4,5]. *O*. *mykiss* is one of the most widely farmed salmonid species owing to its fast growing and its extraordinary adaptation ability [6]. It can resist the hard farming or the wildlife in the surface and the underground water sources of the mountainous regions; however, it is the most susceptible species to ERM disease [7,8].

Effective vaccines had been reported to elevate rainbow trout immunity against ERM disease via oral, bath or injection treatments [9,10]. However, disease control using antibiotics or vaccines leave harmful side effects on fish immunity and environment. Nanoparticles of high antimicrobial activity are currently considered as one of the most advanced and promising avenues to compete usage of antibiotics for disease control in aquaculture [11]. Metal nanoparticles were reported as potential antimicrobials in vitro [12,13], and were able to combat the infections in vivo [14,15]. However, the contemporary trend for fish immunomodulation and disease resistance has been carried out recently using dietary natural supplements and feed additives since they are more safe and applicable in vivo [16]. Therefore, the bio-sourced biodegradable nanoparticles, which are prepared from natural materials and mostly characterized by their low cyto- and genotoxicity [17,18] are considered promising for a wide range of applications in fish medicine.

The marine bio-sourced chitosan nanoparticles (CSNPs) along with the parent chitosan (a natural cationic polysaccharide prepared by the partial alkaline deacetylation of crustacean shells chitin) have low toxicity, anti-microbial activity, mucoadhesivity, and hemocompatibility and are promising for several biomedical applications serving fish aquaculture [19,20]. CSNPs showed in vitro antimicrobial efficacy against several fish bacterial and fungal pathogens [21]. In addition, they are effective feed additives capable of enhancing fish immunity against infectious threats and improving their survival, growth, and meat quality [22,23]. Moreover, CSNPs are effective encapsulators and carriers and can be used for oral delivery of vaccines and bioactive ingredients owing to their high resistant to intestinal degradation. They are able to protect the encapsulated ingredients, extend their shelf life, and enhance their intestinal absorption without cytotoxicity [24,25]. However, more in vivo investigations are still required to evaluate the immunomodulation combined with the antimicrobial efficacy of dietary CSNPs for prevention and/or treatment combating fish diseases.

In an earlier study, dietary chitosan was employed for the immunomodulation of *O. mykiss* and improved its hematological parameters and stress resistance [26]. Additionally, dietary CSNPs was employed for the oral delivery of vitamin C [27,28], and clinoptilolite natural zeolite [29,30] aiming at improving *O. mykiss* growth performance, digestion, intestinal histology, and non-specific immunity. Such studies have assessed the improvement of the systemic immune responses and/or disease resistance; however, fish antimicrobial ability and intestinal immunity still needs more in depth investigation.

We have previously demonstrated the antibacterial efficacy of CSNPs against several fish pathogens, including *Y. ruckeri* [31]. Herein, the current study is aimed at providing knowledge about the impact of dietary CSNPs on the modulation of *O. mykiss* antibacterial defense ability and its intestinal immune status against ERM infection. The effect of short-term CSNPs-feeding trial (21 days) on *O. mykiss* health was evaluated via monitoring fish growth performance and survival, histological traits, antibacterial defense, and intestinal immunity. During a subsequent post-challenge trial, trout disease resistance was evaluated via monitoring the disease associated symptoms and mortality, fish antibacterial defense, and intestinal immunity. The fish antibacterial defence was evaluated in vitro via assessing skin mucus and serum bacterial growth inhibitory activity, and their intestinal immunity was evaluated via assessing histochemical and molecular appraisals. The histochemical appraisal included monitoring the traits in goblet cell count according to their mucin production. The molecular appraisals were assessed by monitoring the relative expression of four intestinal key immunological genes, interleukin 1β (IL1-β), transforming growth factor-β (TGF-β), lysozyme II (LYZ II), and immunoglobulin-M heavy chain (IgM H) in the lower intestine. In addition, the histopathological traits associated with ERM infection were considered. The obtained results provide an insight into the promising role of CSNPs as a therapeutic immunomodulatory feed additive for combating fish disease outbreaks.

## 2. Results

### 2.1. Characterization of CSNPs

#### 2.1.1. Particle Size and Size Distribution

The nanoparticles mean size along with the particle size distribution pattern of CSNPs prepared during the current study are displayed (Supplementary Materials, Figure S1). The figure shows mean particles diameter of 100 nm (Supplementary Materials, Figure S1a). The range of particles size distribution is narrow and rages from 73 to 145 nm (Supplementary Materials, Figure S1b) and a polydispersity index of 0.5.

#### 2.1.2. Particle Shape

The scanned particles of the prepared CSNPs appeared regular spherical in shape under the scanning electron microscopy (SEM) and no agglomerations was detected (Supplementary Materials, Figure S2a). The transmission electron microscopy (TEM) showed regular surfaces and homogenous size of the spherical particles (Supplementary Materials, Figure S2b). The diameter range of the scanned particles was 70.23–159.53 nm and their average is 103.22 nm.

#### 2.1.3. CSNPs Crystalline Structure

X-ray diffraction (XRD) spectra of chitosan and chitosan nanoparticles are shown in (Supplementary Materials, Figure S3). Chitosan has two prominent crystalline peaks at 2θ = 10° and 21.8° (Supplementary Materials, Figure S3a), which are in agreement with Samuels’ report [32]. It was observed that there was a remarkable decrease in the intensity of the characteristic peak for CSNPs (chitosan cross-linked with sodium tripolyphosphate (TPP)). This finding may be due to the arrangement modification of the molecules in the crystal lattice during the process of cross linking, which is in a good accordance with the previously reported data [32,33,34].

### 2.2. Oncorhynchus mykiss Health after Three Weeks Feeding Trial

#### 2.2.1. Growth Performance and Fish Survival

Regardless of the treatment, fish body weight was enhanced after three weeks of *ad libitum* feeding in comparison to the initial weights recorded at zero time-point sampling before starting the feeding trial. In spite of the very high significant increase (*p* < 0.001) in the final fish weight, no significant differences (*p* > 0.05) were detected after this short-term feeding period in terms of the body weight gain or the specific growth rate in comparison with the non-treated control group. Additionally, short-term supplementation of CSNPs in diets was not a significant factor (*p* > 0.05) affecting fish survival, which was kept at 100% in all tanks. Growth performance and fish survival data were expressed as means ± SD and displayed in Table 1.

All data are expressed as mean ± SD (n = 18/treatment). Different superscript letters on the mean values of a column indicates the statistically different data (*p* < 0.05). Weight gain (WG) = 100 × [(Final body weight (FBW) − Initial body weight (IBW))/(Initial body weight (IBW))]. Specific growth rate (SGR) = 100 × [(Ln final body weight (FBW) − Ln initial body weight (IBW))/(number of days of the feeding trial (21))]. Survival = 100 × [(Final number of fish)/(Initial number of fish)].

#### 2.2.2. Intestinal Histological Appraisals

The histological appraisals of *O. mykiss* anterior intestine after three weeks CSNPs treatment were examined on triplicate micrographs from Hematoxylin & Eosin (H&E) counter staining cross-sections per group. No obvious histological differences were observed between the non-treated intestine sampled at zero time-point or at the 21st day post feeding (dpf). Short-term CSNPs treatment was not a factor affecting fish intestinal villi length, where no statistically significant increase (*p* > 0.17) was observed on villi length of CSNPs-treated anterior intestinal mucosa in comparison with the non-treated control intestinal samples. The average villus length recorded 364.60 ± 56.43 and 358.57 ± 48.51 µm (n = 15) at zero time-point, and 366.07 ± 58.5 and 396.79 ± 61.14 µm (n = 15) for the non-treated control and CSNPs-treated intestinal samples, respectively.

### 2.3. Oncorhynchus mykiss Resistance against ERM Infection

#### 2.3.1. Skin Mucus and Serum Antibacterial Defense

Three weeks dietary CSNPs revealed an improvement in *O. mykiss* antibacterial defense against the pathogenic bacterium *Y. ruckeri*. Skin mucus of CSNPs-treated trout showed wider zones of bacterial growth inhibition (ZOI), and lower minimum inhibitory concentration (MIC) values than the skin mucus sampled before treatment or on the 21st dpf from the non-treated fish. Statistically, the skin mucus ZOI of CSNPs-treated trout were very significantly increased (*p* < 0.001) in comparison with trout received non-supplemented feed during the short-term feeding trial. Mean measurements of the skin mucus ZOI and MIC after the short-term feeding trial are collected in Table 2.

ZOI data are expressed as mean ± SD (n = 9/treatment). Different superscript letters on the mean values of a column indicates the statistically different data (*p* < 0.05). MIC value of the three replicates per group that was repeated twice, or more was considered.

Opposed to the non-infected fish, there was an increase in the skin mucus antibacterial defense of all the challenged trout up to the 10th day post infection (dpi). MIC values get lowered, and a significant increase (*p* < 0.001) in the skin mucus ZOI was observed on all the challenged groups. Reaching the 28th dpi, skin mucus antibacterial defense was higher than the non-treated, non-infected fish only in *O. mykiss* treated with CSNPs by therapeutic-regimen. MIC value was the lowest of all groups, and the mean ZOI was significant increased (*p* < 0.01). Whereas no considerable difference was observed on the skin mucus antibacterial activity between the rest of challenged groups and the pathogen-free control group. Obviously, CSNPs-treated fish by therapeutic-regimen elicited the lowest MIC values, and the highest significant increase in the skin mucus ZOI of all groups at all the sampling time-points. Table 3 displays the skin mucus antibacterial activity results, including ZOI and MIC values recorded throughout the post-challenge trial.

ZOI data are expressed as mean ± SD (*n* = 9/treatment). Different superscript letters on the mean values of a column indicates the statistically different data (*p* < 0.05). MIC value of the three replicates per group that was repeated twice, or more was considered.

**Figure 1:**
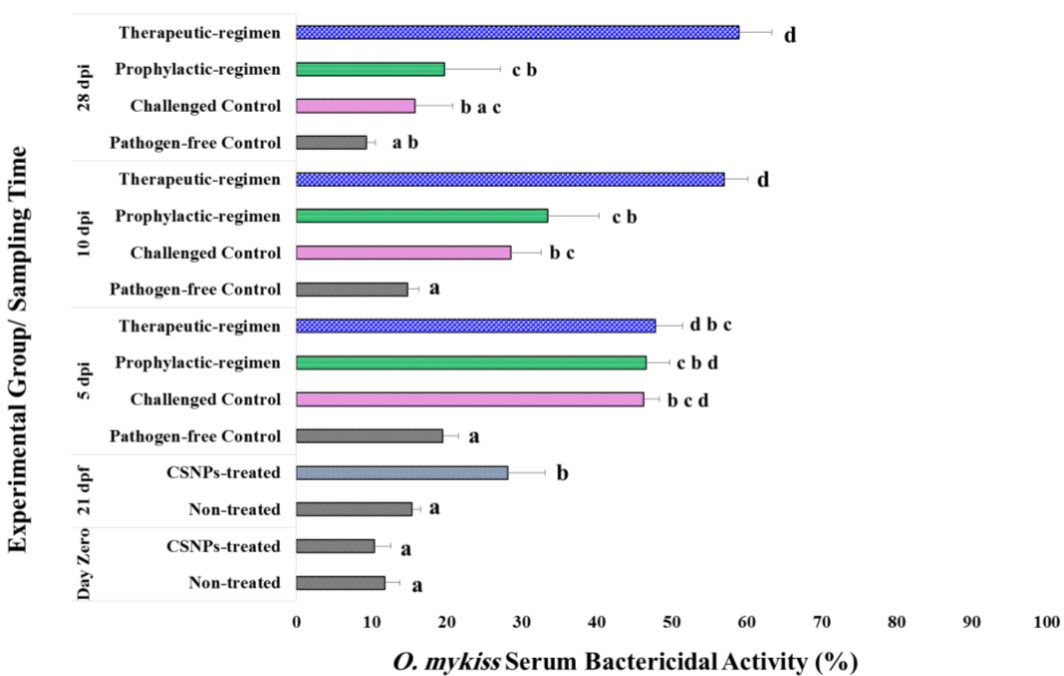
Serum bactericidal activity (%) against the virulent isolate *Y. ruckeri* (CSF007-82) elicited by *O. mykiss* fed on non-supplemented diets and diets supplemented with CSNPs (5g kg^−1^ dry feed weight) by two different regimens throughout the pre- and post-challenge trials. Data are presented as mean ± SD (*n* = 9/treatment). Different superscript letters on columns indicates the statistically different data between groups (*p* < 0.05).

Similarly, three weeks treatment with CSNPs enhanced the trout serum antibacterial defense (%) against *Y. ruckeri* as shown in Figure 1. Mean value of the bacterial growth inhibition percent elicited by CSNPs-treated fish sera was higher than that was elicited by the sera sampled before treatment or sampled from the non-treated fish at the 21st dpf. Statistically, serum bactericidal activity of CSNPs-treated trout increased significantly (*p* < 0.001) in comparison with the activity of sera from trout receiving non-supplemented feed over a three-week feeding trial.

During the post-challenge trial, CSNPs-treated fish by therapeutic-regimen showed the highest significant increase in terms of serum bactericidal activity at all the sampling time-points. Very high significant increase (*p* < 0.001) in the serum bactericidal activity of all the challenged trout was shown up to the 10th dpi. No statistically significant differences were recorded between the challenged groups on the 5th dpi. Whereas on the 10th dpi, serum bactericidal activity of CSNPs-treated fish by therapeutic-regimen was significantly higher (*p* < 0.05) than the other two challenged groups, which did not show significant differences between them. Reaching the 28th dpi, both of the CSNPs-treated groups showed significantly higher (*p* < 0.005) serum bactericidal activity than the pathogen-free control group. Whereas, no statistical difference (*p* > 0.005) was recorded between the CSNPs-treated group by prophylactic-regimen and the non-treated challenged control group. Interestingly, significant increase (*p* < 0.01) in the serum bactericidal activity was elicited from the therapeutic-regimen CSNPs-treated group in compare to the prophylactic-regimen group. Figure 1 displays serum bactericidal activity of *O. mykiss* throughout the feeding as well as the challenge trials, which refers to higher resistance against ERM infection from the trout treated with CSNPs by therapeutic-regimen.

#### 2.3.2. Intestinal Immunity Investigations

I. Histochemical Characterization and Semi-Quantification of Mucin-Producing Goblet Cells

**Figure 2:**
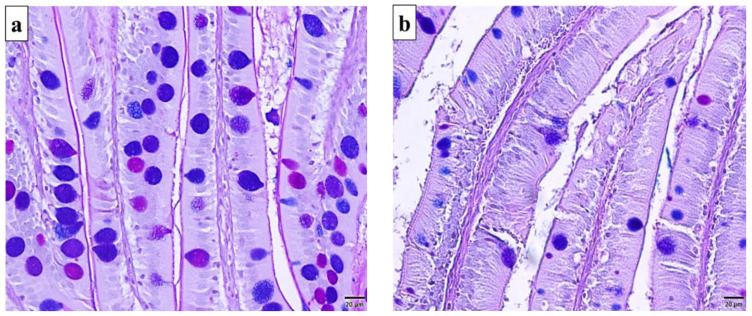
Photomicrographs of AB & PAS double stained cross-sections from *O. mykiss* anterior intestines displaying the histochemical characterization of goblet cell types in *O. mykiss* anterior intestine before and during ERM acute infection (i.e., 5th day post infection) (×400). (**a**) Pathogen-free intestine showing more frequent mucin-filled goblet cells that are positively stained blue, pink, or purple; (**b**) ERM infected intestine showing more frequent negatively stained mucin-free goblet cells associated with shedding out of the villi epithelium.

The histochemical traits of *O. mykiss* anterior intestine were examined in triplicate micrographs of Alcian Blue & Periodic-Acid Schiff (AB&PAS) doubled staining cross-sections per group. Histochemical characterization of goblet cells in the pre-challenge anterior intestinal samples, including the pre-treatment samples revealed more frequent mucin-filled goblet cells than the empty negatively stained mucin-free goblet cells (Figure 2a). On the contrary, the infected samples on the 5th dpi, which is the acute infection time, showed more frequent mucin-free goblet cells (Figure 2b) than the other pathogen-free samples. On the other hand, mucin-filled goblet cells returned more frequent than the mucin-free cells on both the 10th dpi and the 28th dpi. This may indicate the intensive mucus secretion as an intestinal washing up strategy during the intensive pathogen invade.

**Figure 3:**
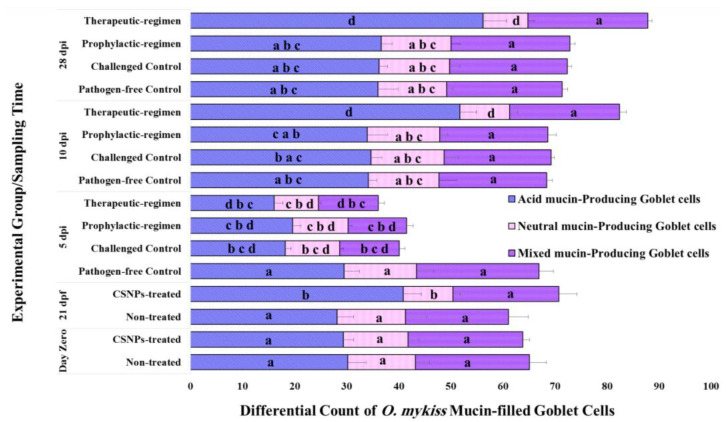
Effect of dietary CSNPs (5g kg^−1^ dry feed weight) on the differential count of mucin-filled goblet cell types within 1000 µm mucosal epithelial length of *O. mykiss* anterior intestine before and after the ERM infection. Data are presented as mean ± SD of the count within triplicate cross-sections per group (1 fish per tank) through triplicate fields per section (*n* = 9). Different superscript letters on similar columns sections of each time-point indicates the statistically different data between groups (*p* < 0.05).

Differential count of goblet cells in the pre-challenge and post-challenge anterior intestinal samples at all the sampling time-points of our experiment is displayed in a column histogram (Figure 3). After 21 days feeding trial, higher count of acid mucin-producing goblet cells was observed on CSNPs-treated anterior intestinal samples than the non-treated or the pre-treatment samples. Very high significance (*p* < 0.001) increase in the acid mucin-producing goblet cells count, and decrease in the neutral mucin-producing cells count was recorded in CSNPs-treated samples in comparison with the non-treated control group. Whereas, CSNPs had no effect on the mixed mucin-producing goblet cells since no significant difference (*p* > 0.12) was recorded between groups in terms of their count. As for the post-challenge samples, no significant difference (*p* > 0.52) was observed between the challenged groups in terms of the total and the differential count of their mucin-filled goblet cell content on the 5th dpi. In addition, the total count of mucin-filled goblet cells of the pathogen-free control group was the highest (see Figure 3). This may be due to the excessive mucus secretion from the challenged intestines at that time-point. On the 10th and the 28th dpi, the therapeutic-regimen CSNPs-treated group recorded the highest total count of mucin-filled goblet cells of all groups. In addition, this group recorded the significantly highest (*p* < 0.05) number of acidic mucin-producing goblet cells, and the significantly lowest (*p* < 0.05) number of the neutral mucin-producing goblet cells of all groups (see Figure 3). At these time-points, no significant differences were observed between the other groups in terms of goblet cells total or differential count. Regarding mixed mucin-producing goblet cells count, no significant difference (*p* > 0.09) was recorded during the post-challenge trial between the challenged groups or in comparison to the non-treated control group, except for the 5th dpi, which showed significant higher number of the non-treated samples (see Figure 3). Our results refer to the higher intestinal antibacterial components during dietary CSNPs, especially with the therapeutic-regimen.

II. Relative Expression of Some Intestinal Immune-Relevant Genes

**Figure 4:**
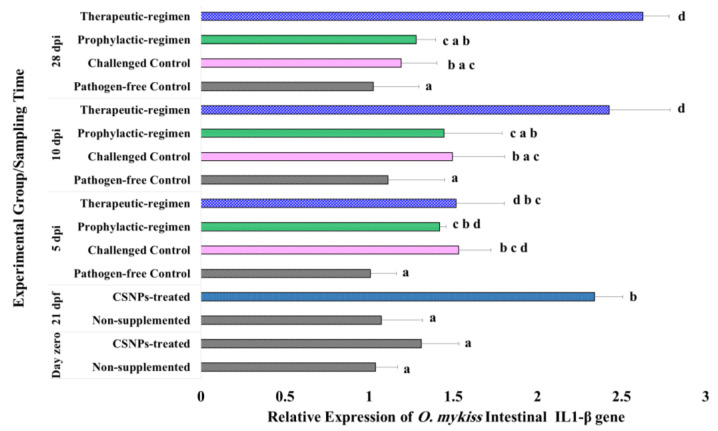
Expression pattern of IL1-β gene relative to both β-actin and EF1-β reference genes in the lower intestine of *O. mykiss* fed with CSNPs supplemented or non-supplemented diets, before and after *Y. ruckeri* challenge. Data are presented as mean ± SD of duplicate technical replicates from triplicate biological replicates per group (*n* = 9). Different superscript letters on columns of each time-point indicates the statistically different data between groups (*p* < 0.05).

**Figure 5:**
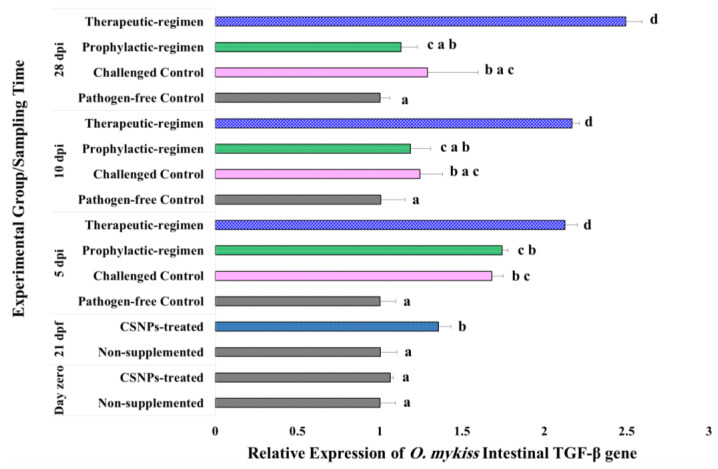
Expression pattern of TGF-β gene relative to both β-actin and EF1-β reference genes in the lower intestine of *O. mykiss* fed with CSNPs supplemented or non-supplemented diets, before and after *Y. ruckeri* challenge. Data are presented as mean ± SD of duplicate technical replicates from triplicate biological replicates per group (*n* = 9). Different superscript letters on columns of each time-point indicates the statistically different data between groups (*p* < 0.05).

**Figure 6:**
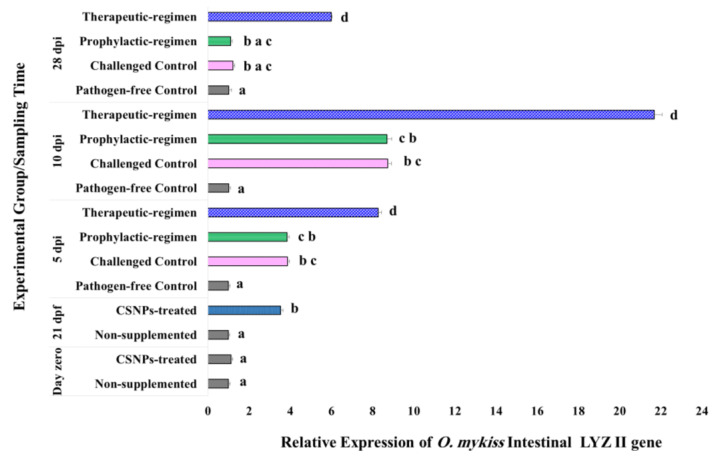
Expression pattern of LYZ II gene relative to both β-actin and EF1-β reference genes in the lower intestine of *O. mykiss* fed with CSNPs supplemented or non-supplemented diets, before and after *Y. ruckeri* challenge. Data are presented as mean ± SD of duplicate technical replicates from triplicate biological replicates per group (*n* = 9). Different superscript letters on columns of each time-point indicates the statistically different data between groups (*p* < 0.05).

**Figure 7:**
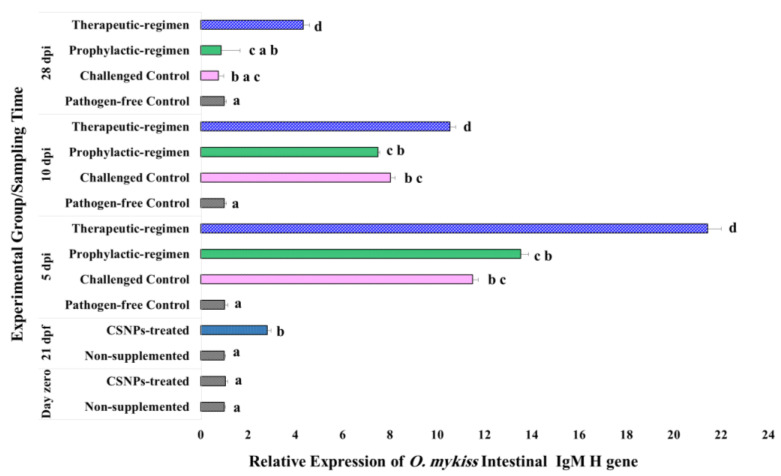
Expression pattern of IgM H gene relative to both β-actin and EF1-β reference genes in the lower intestine of *O. mykiss* fed with CSNPs supplemented or non-supplemented diets, before and after *Y. ruckeri* challenge. Data are presented as mean ± SD of duplicate technical replicates from triplicate biological replicates per group (*n* = 9). Different superscript letters on columns of each time-point indicates the statistically different data between groups (*p* < 0.05).

*O. mykiss* intestinal molecular traits after feeding with CSNPs were investigated on the relative expression of four target immune-relevant genes in the lower intestinal samples of the pre-challenge as well as the post-challenge groups. The obtained data were presented in four column histograms (Figure 4, Figure 5, Figure 6 and Figure 7) displaying the relative expression of each target gene throughout both trials. After the short-term feeding trial (21 days), the expression of IL1-β and TGF-β genes of the CSNPs-treated samples showed statistically significant up-regulation (*p* < 0.05) in relative to the non-treated control samples. The relative expression of LYZ II and IgM H genes also augmented, however the difference was not statistically significant (*p* > 0.05).

In response to the bacterial challenge, therapeutic-regimen CSNPs-treated samples kept the significant increase (*p* < 0.04) in the expression of all the target genes at all the sampling time-points relative to the pathogen-free control samples. Similarly, the expression of all the investigated genes increased significantly (*p* < 0.05) on the 5th dpi for the prophylactic-regimen CSNPs-treated, and the non-treated challenged control samples as compared with the non-treated pathogen-free control samples. On the 10th dpi, the relative expression of both LYZ II and IgM H genes in the prophylactic-regimen CSNPs-treated, and the non-treated challenged control samples was kept significantly higher (*p* < 0.05), and an augmentation with no statistically significant difference was observed on the expression of both TGF-β and IL1-β genes. By the end of experiment (on the 28th dpi), the expression of all the investigated genes of both groups was up-regulated relative to the non-treated free-pathogen control samples with non-significant down-regulation of LYZ II and IgM H gene expression. Our results showed higher intestinal immunity after dietary CSNPs, and higher anti-bacterial and anti-inflammatory avidity of the therapeutic-regimen.

### 2.4. Clinical Signs Associated with ERM Disease Progression

#### 2.4.1. Symptoms and Mortality 

**Figure 8:**
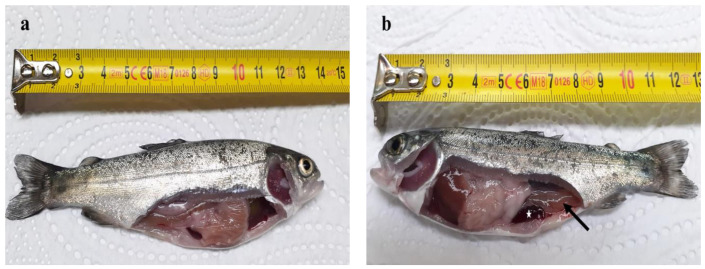
Photomicrograph displaying the symptomatic macroscopic signs of ERM infection on *O. mykiss* internal organs. (**a**) Control unchallenged fish; (**b**) ERM infected fish showing enlarged darkened spleen (white star), and reddened lower intestine filled with yellowish fluids (black arrow).

Two days post-challenge, common symptoms associated with the ERM infection were observed on the experimentally infected fish in the form of poor feeding response and swimming near tanks edges or surfaces. Furthermore, signs of skin darkness, hemorrhagic congestions of tails, and exophthalmia were observed on both the challenged-control group, and the CSNPs-treated group by prophylactic-regimen. In addition, complete loss of appetite was observed on the challenged-control fish. No further clinical signs were observed on the group treated with CSNPs by therapeutic regimen, which indicates higher disease resistance. Upon reaching the 28th dpi, all the remaining infected fish could recover, and no further clinical signs were recorded in all groups. During dissection, no obvious differences were noticed between the infected fish of all groups in terms of the clinical signs of the internal organs. Macroscopically, the observed clinical signs were in the form of darkened enlarged spleens, and reddish lower intestine filled with yellowish fluid (Figure 8).

**Figure 9:**
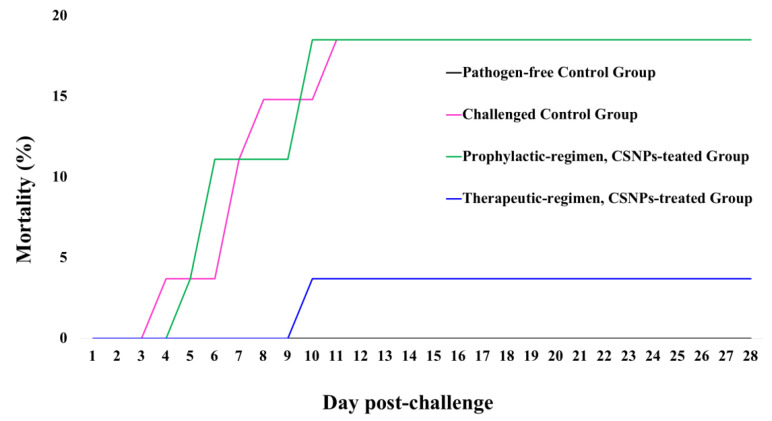
The cumulative mortality of *O. mykiss* fed on non-supplemented diets or diets supplemented with CSNPs (5g kg^−1^ dry feed weight) by two different regimens over 28 days post-challenge with *Y. ruckeri* (CSF007-82). Data are presented as the mean ± SD (the fish sampled during the post-challenge trial were counted as surviving fish).

Throughout two symptomatic weeks, the disease progression was associated with moribund fish starting from the 4th dpi until the 12th dpi. During these eight days, a total of 11 moribund fish were counted as mortality; therefore, fish cumulative mortality (%) was assessed in Figure 9. The relative percent survival (RPS) of CSNPs-treated groups recorded 80% for the therapeutic-regimen and 0% for the prophylactic-regimen groups in comparison with the challenged control group.

#### 2.4.2. Histopathological Appraisals 

**Figure 10:**
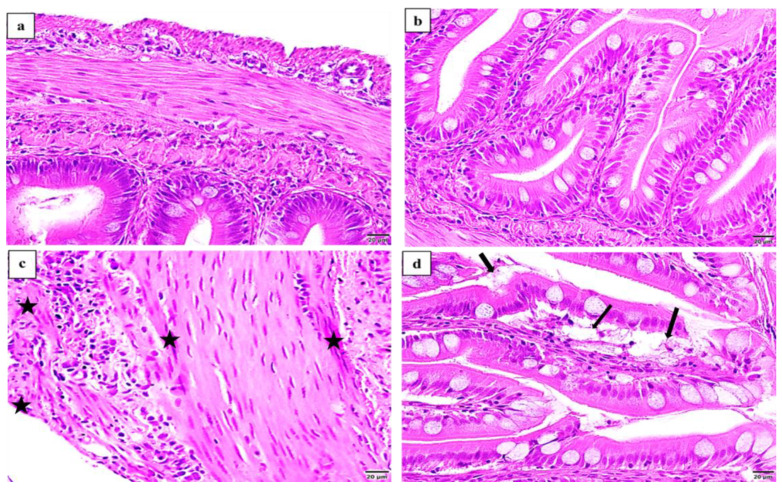
Photomicrographs of H&E counterstained cross sections from *O. mykiss* anterior intestines displaying the most common histopathological traits associated with ERM infection (× 400). Pathogen-free anterior intestine showing (**a**) intact muscularis; (**b**) intact villi. Infected anterior intestine showing (**c**) congestions of serosa and muscularis (stars); (**d**) shedding out of the mucosal epithelium (arrows).

No considerable markers of intestinal histopathological resistance were observed in comparison between the challenged groups. The histopathological traits association with ERM infection were in the form of congestions of serosa and muscularis and shedding out of the mucosal epithelium (Figure 10).

## 3. Discussion

Short-term feeding with CSNPs (5 g kg^−1^ feed) for 21 days did not affect *O. mykiss* growth performance or its intestinal histological structure. No significant traits were observed on the villus length of CSNPs-treated *O. mykiss* anterior intestines compared to the non-treated control fish samples. In contrary, 60 days of feeding with CSNPs (5 g kg^−1^ feed)/zeolite composite could improve the histological structure of *O. mykiss* entire intestine, including the villus height, density and absorption surface area [34]. Our findings agreed with Abd El-Naby et al. [35] who reported normal anterior intestinal villus length of Nile tilapia fingerlings fed with CSNPs (5 g kg^−1^ feed) for 70 days. On the other hand, our results disagreed with Abd El-Naby et al. [36] who reported a significant increase in the mid-gut villi length of Nile tilapia fingerlings fed for 70 days on CSNPs (5 g kg^−1^ feed) mixed with the phenolic essential oil ‘Thymol’. However, normal appearance with a significant increase in the length of the anterior intestinal villi of Caspian kutum fingerlings were reported upon 60 days feeding with chitosan [37].

Fish skin mucus and sera contain natural antibodies and protective proteins incorporated with fish non-specific immunity, which enable them to elicit a strong bactericidal activity against pathogens [38,39]. Therefore, they both are able to inhibit the bacterial growth in vitro, and fish skin mucus was reported to elicit a stronger bactericidal activity than serum [40]. Diets supplemented with bio-sourced immunostimulants could promote fish skin mucosal immunity and bactericidal activity against pathogenic and non-pathogenic bacteria [41,42]. Our results are in the same line with these studies and showed bactericidal activity from healthy *O. mykiss* skin mucus against the pathogenic *Y. ruckeri* isolate, with significantly higher bactericidal effect after receiving CSNPs (5 g kg^−1^ dry feed weight) for 21 days. In a previous study, *O. mykiss* skin mucus antibacterial activity was promoted against the pathogenic *Y. ruckeri* by receiving diets supplemented with the immunostimulant, Ergosan, obtained from brown sea weeds for 50 days [43]. *O. mykiss* serum bactericidal activity was enhanced against *Y. ruckeri* after receiving diets supplemented with decaffeinated green tea extract for 30 days [44]. In the current study, the bacterial challenge with the pathogenic *Y. ruckeri* promotes the trout skin mucus and serum bactericidal activities in comparison with the pathogen-free control fish against the same isolate at the same challenge concentration. Similarly, higher skin mucus and serum bactericidal activities were elicited by the challenged fish in comparison with the pathogen-free fish samples [39,45]. It is worth mentioning that during our study the mucus as well as serum antimicrobial activity assays were proceeded on the freshly collected samples to avoid decreasing their humoral immune activity levels by freezing at −20 °C or −80 °C, or by lyophilization [46].

Goblet cells are the most dominant mucus-producing cell type within fish intestinal mucosa. *O. mykiss* intestinal mucus contains immune complement components and antimicrobial peptides incorporated in its innate immunity and plays an important role as a barrier against pathogens [47,48]. The most predominant contents of fish intestinal mucus are the viscous glycoproteins, mucins, which have different types according to their glycosylation degree, and consider the main site of mucus interaction with the surroundings in addition to granting its antimicrobial characteristics [49,50]. The intestinal mucins contain complex glycans can act for pathogens elevation from the internal epithelium [51]. However, the commensal intestinal bacteria produce proteases that can rapidly degrade the immune elements of the intestinal mucus after being secreted in the lumen hindering their accurate quantification [49]. Therefore, AB&PAS histochemical double staining is employed for the semi-quantification of the intestinal villi mucin-producing cells, which provides an insight on the intestinal immunity. Two types of intestinal mucin-producing goblet cells (neutral and acidic) were previously identified and characterized histochemically in rainbow trout [52], additionally, their dynamics have been investigated after different dietary conditions [34,53]. Moreover, the protective response of fish intestinal mucus-producing cells have been investigated after parasitic infection cases [54,55], and after a combination of dietary and bacterial intubation conditions [56]. Herein, our study provides an investigation of *O. mykiss* gut mucin dynamics after 21 days dietary CSNPs as well as their protective response dynamics against the ERM infection. Our results revealed more frequent mucin-filled goblet cells than the mucin-free cells in all the pre-challenged samples. Of them, the count of acid mucin-producing cells was significantly higher, and the count of neutral mucin-producing cells was significantly lower in CSNPs-treated group than in non-treated group. This is in an agreement with Hamidian et al. [34] who reported significantly higher percentage of acid mucin-producing cells, while lower neutral mucin-producing cells in the anterior intestine of *O. mykiss* fed with CSNPs (5 g)/zeolite (14.28 g)/kg supplemented feed for 60 days. Conversely, the count of neutral mucin-producing goblet cells was significantly higher in *O. mykiss* anterior intestine after insect protein meal [53]. However, acid and neutral mucin-producing goblet cells were present equally in *O. mykiss* gut sampled from several fish culture ponds [52]. This refers to the effect of feed composition on fish gut mucin production. 

In the current study, after challenge, a significant reduction was recorded on mucin-filled goblet cells of all the challenged groups on the 5th dpi, while no significant difference was recorded on the 10th and the 28th dpi in comparison with the non-treated pathogen-free control. This might refer to the intestinal mucus production, secretion, and reproducing kinetics during the bacterial invasion where the intestinal mucus is the first gut defence line against pathogens’ invasion. On the 10th and the 28th dpi, a significant increase was recorded on the count of acid mucin-producing goblet cells of the therapeutic-regimen, CSNPs-treated group. It is known that acid mucins (sulfated) are resistant against the intestinal and bacterial degradation providing higher protection to the intestinal epithelium. Therefore, shifting the mucin-producing cell type to the acidic, which is indicated by their higher quantification, reinforced higher intestinal mucosal immunity [34]. Similar fluctuation in the number of mucin-filled goblet cells as dynamics between gut mucus secretion and pathogen invade was reported in common carp anterior intestine fed with β-glucan after few days up to 7 days of its oral intubation with *Aeromonas hydrophila* [56]. The author returned the reason to the intestinal defense against pathogens via the excessive release of mucus containing acid mucins, which bind with the bacteria and therefore washing them out of the gut.

Regarding the intestinal immune-relevant genes targeted by our study, it is known that IL1-β, TGF-β, IgM, and LYZ encode immune-relevant genes that provide different immune response avidities, which in turn activates fish innate and adaptive immunity. IL1-β is a pro-inflammatory cytokine encoding IL interleukin-1β peptide chain, which provides an early or on-time immune response via lymphocytes activation or the promotion of releasing several immune-relevant cytokines [30,57,58]. For example, *O. mykiss* IL1-β-derived protein could stimulate macrophages for higher in vitro phagocytosis and bactericidal defence against *A. salmonicida* [59]. TGF-β is an anti-inflammatory cytokine having a role in initiating the inflammatory reactions for wounds visualisation, hematopoiesis, and healing via stimulating the rapid accumulation of macrophages, granulocytes, and other cells at the wound site, or sustain acute inflammations in combination with other cytokines [60]. It can induce the mucosal tissues for a positive immune tolerance based on its deep effect on the immune cells (lymphocytes and macrophages and dendritic cells) [57]. IgM encoding gene is involved in the antibody production where it is encoding the immune-related protein ‘immunoglobulin’, which is present in the serum and the mucosal secretions and involved in systemic immune responses and gut adaptive immunity [49]. However, IgM protein might be detected in blood by higher levels than the gut because of its fast degradation by gut mucus proteases, which in turn hinders its accurate quantification and qualification analysis [49]. LYZ encoding gene is involved in gut innate immunity and can stimulate lysozyme production for the antibacterial defence purposes [61,62].

As for the relative expression of the intestinal immune-relevant genes, immune cell biomarkers for gut adaptive immune responses are expressed in mid and hind gut more than the anterior intestine or stomach. All our target genes were up-regulated following 21 days dietary CSNPs, of them, IL1-β and TGF-β showed statistically significant expression. Our results agree with a previous report where dietary CSNPs/clinoptilolite mixture significantly increased the relative expression of immune-relevant genes in *O. mykiss* head kidney after a 72-day feeding period. The expression of IL1-β was up-regulated by 0.05, 0.5 or 5g kg^−1^ feed doses of CSNPs, while the expression of IgM was up-regulated by a 0.05 g kg^−1^ feed dose [30]. In a similar context, several immunostimulant feed supplementations improved the expression of *O. mykiss* immune-relevant genes. Significantly higher expression of the intestinal IL1-β, TGF-β, and LYZ genes was reported after 45 days dietary supplementation with sodium butyrate and feed additive [61]. The dietary probiotic bacteria *Lactobacillus rhamnosus* up-regulated the expression of IL1-β, TGF-β, and IgM genes in kidney and spleen; where the expression of TGF-β was augmented and that of IL1-β was increased significantly after 45 days feeding period [63], while TGF-β and IgM relative gene expression was increased significantly after 30 days administration period [64]. Long-term (95 days) dietary administration of the algal extract Ergosan significantly increased the expression of IL1-β gene in the liver of vaccinated juveniles against ERM disease [65]. Short-term (2 weeks) dietary administration of immunomodulatory plant products increased the expression of IL1-β and TGF-β genes in the head kidney. Significantly higher expression of TGF-β gene was reported after feeding with mango (1%), and both of the IL1-β and TGF-β genes were expressed by significantly higher level after feeding with lupin (1%), or stinging nettle (1%, and 2%) [57]. The relative expression of LYZ type c, and TNFα immune related genes was increase significantly in head kidney after 15- against a pathogenic *A. salmonicida* isolate, while the situation was not the same in response to the formalin-killed or the non-pathogenic isolates [66]. In the same context, significant augmentation in the relative expression of IL1-β and TGF-β genes was and 45-days administrations of prebiotic immunogen^®^ (2 g kg^−1^) [67].

After 5 days of ERM infection, the expression of the targeted immune-relevant genes was found to be significantly up-regulated in all the challenged CSNPs-treated and non-treated samples relatively with the pathogen-free control intestinal samples. This might refer at intestinal innate immune response against the invading *Y. ruckeri*, especially during the acute infection condition (one day after the mortality started). Only the therapeutic-regimen, CSNPs-treated group could keep this high relative expression up to the 28th dpi. With regard to the other two groups, significant higher expression of LYZ II and IgM H genes was observed on the 10th dpi, and the expression of all genes was unregulated on the 28th dpi. Similarly, higher expression of IL1-β was reported in the spleen of *O. mykiss* infected with *Y. ruckeri* [68]. Interestingly, IL1-β gene was highly expressed in *O. mykiss* intestine as an immune defence reported in vitro on *O. mykiss* head kidney leucocytes, and on isolated gut cells after co-culturing with the pathogenic *Y. ruckeri* for 6 h and 12 h, respectively, and the situation was not the same in the response of isolated gut cells to the probiotic bacteria [69]. These studies provide an indication on the role of dietary immunostimulants in enhancing the selective intestinal immunity against the pathogenic bacteria, not against the commensals.

As for the intestinal histopathological traits associated with ERM infection, the infected fish showed congestions in serosa and muscularis, and shedding out of the villi epithelium as usual signs of ERM disease reported elsewhere [70,71]. In spite of the mild clinical signs and the very high significance lower mortality observed on the therapeutic regimen CSNPs-treated group, the intestinal histopathology did not provide an obvious parameter for distinguishing the intestinal resistance between CSNPs-treated and the non-treated fish.

## 4. Materials and Methods

### 4.1. Preparation of CSNPs

**Scheme 1:**
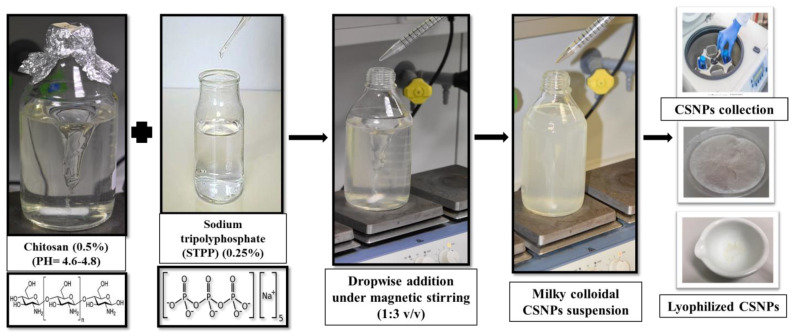
Representative steps that were performed in our labs for CSNPs preparation following the ionic gelation method.

Low molecular weight chitosan (50 kDa, 90% deacetylation degree), sodium TTP salt, acetic acid, and sodium hydroxide (NaOH) (Sigma-Aldrich, Vienna, Austria) were used for the preparation of CSNPs. They were prepared following the ionic gelation method [72] as shown in Scheme 1. In brief, clear chitosan ionic solution (0.5%, *w/v*) was prepared in dilute aqueous HOAc (1%, *v/v*) via continuous magnetic stirring, and its pH was raised to 4.6–4.8 via NaOH (10 N). titration. CSNPs were prepared by dropwise adding of an aqueous TPP solution (0.25%, *w/v*) into the chitosan solution by a ratio of 1:3, under continuous magnetic stirring at room temperature. Milky colloidal CSNPs suspension was formed after 2 h of ionic crosslinking interaction between the two oppositely charged ions. The CSNPs were precipitated and collected via 4,000 × g centrifugation for 30 min at room temperature. Three times washing in distilled water with extensive rinsing was performed for CSNPs purification. Then, the purified CSNPs were again collection via further 4,000 × g centrifugation for 30 min at room temperature. The collected gel-like colloids of CSNPs were lyophilized and kept at 4 °C for further use or analysis.

### 4.2. Characterization of CSNPs

#### 4.2.1. Particles Size and Size Distribution

Malvern Zetasizer Nano ZS^®^ device of dynamic light scattering was used for the estimation of the particle size and the size distribution of the obtained CSNPs. Diluted CSNPs suspension in de-ionized distilled water was analysed in triplicate under 90° scattering angle at 25 °C [31].

#### 4.2.2. Electron Microscopy

Particle shape, surface morphology, aggregations, and average size of the prepared CSNPs were monitored by usual electron microscopy assay. The microscopy was performed in triplicate on diluted suspensions of the freez-dried CSNPs powder. SEM (Phillips-500, Hamburg, Germany) was used for assessment of the particle shape and aggregates. TEM (EM 900, Zeiss, Oberkochen, Germany) was used for assessment of the morphology and average diameter of phosphate tungsten acid negatively stained particles.

#### 4.2.3. X-ray Diffraction

The XRD patterns of chitosan and CSNPs (crosslinked chitosan with sodium TPP) were measured using an x-ray diffractometer (PW 1729, Philips, Eindhoven, Netherlands). The samples were irradiated with mono-chromatized Cu Kα radiation (1.542 Å) and analyzed between 2 and 60 (2 θ). The voltage and current used were 30 kV and 30 mA, respectively. The range and the chart speed were 2 × 10^3^ CPS and 10 mm (2 θ°)^−1^, respectively.

### 4.3. Diet Formulation

Medicated diet containing CSNPs (5g kg^−1^ dry feed weight) was prepared according to Alishahi et al. [27] with some modifications. The safety and effectiveness of this high dose in diets was approved previously by Wang et al. [22] and was frequently applied and known by its positive impact on fish immunomodulation and intestinal structural status [28,29,34,35,36]. Powdered CSNPs were weighed (Xs Balance mod. 224–220gr. −0.1 mg) and mixed thoroughly with rainbow trout commercial feed, which was soften by soaking in a minimum amount of deionized water for 30 min. The medicated feed mixture was re-pelleted using an extruder machine having small holes of 1 mm. The feed pellets were dried thoroughly at room temperature (29 ± 1 °C) and stored in firmly closed polypropylene bags at −20 °C until further use [22,73].

### 4.4. Fish Rearing and Experimental Conditions

The current study was performed at the experimental facility of Department for Farm Animals and Veterinary Public Health, Clinical Division of Fish Medicine, University of Veterinary Medicine, Austria. Procedures for animal care and management were conducted according to the guidelines of the European institutional ethics and animal welfare after the approval from the Ministry of Science, Austria according to §26ff of the Austrian laws for care and use of experimental animals (GZ: 2020-0.001.578).

Healthy *O. mykiss* were purchased from a certified category one local fish farm and kept for acclimation in our wet laboratory. The average total body weight was 15.53 ± 0.67 g, and the average total body length was 11.06 ± 0.46 cm (means ± standard deviation (SD)). Fish were kept in 75 L capacity tanks supplied with flow-through system (0.5 L s^−1^), adequate aeration (9 ± 0.5 ppm dissolved oxygen), and electric rod heaters maintaining the water temperature at 16 ± 2 °C. The water quality parameters including, temperature, dissolved oxygen, and pH were surveyed daily in each tank, while the total hardness, carbonate, nitrate, and nitrite levels were surveyed once a week. Fish were acclimated for 14 days and fed twice daily *ad libitum* on commercial feed pellets (Aqua Dynamic Semi Swim/2, Garant Aqua), and exposed to a 12 h light-dark cycle.

### 4.5. Fish Disease Status 

Upon fish arrival and at each sampling time-point, fish were checked for the pathogen-free status. Samples of brain, kidney, and spleen were spread on blood agar (BA) plates (Sigma-Aldrich, Austria) [14]. In addition, intestines collected from moribund or sampled fish throughout the post-challenge trial were squeezed in sterile tryptone yeast extract media (Sigma-Aldrich, Austria), and the fecal contents were spread on BA plates. The inoculated agar plates used to be incubated at 20 °C for 10 days and observed regularly for *Y. ruckeri* growth [74].

### 4.6. Experimental Design

**Scheme 2:**
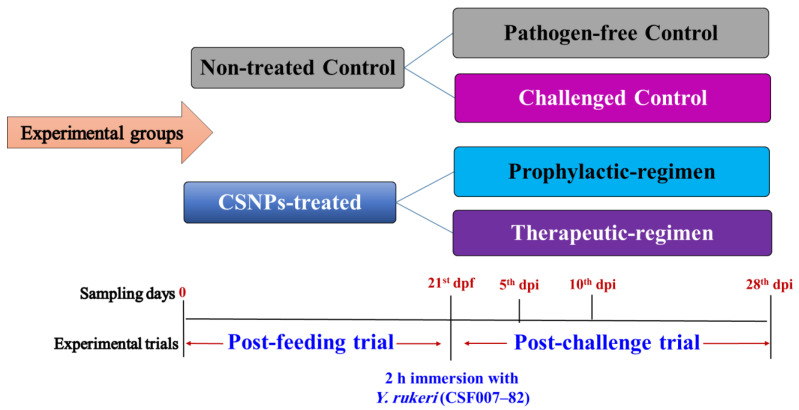
Experimental design and fish sampling time-points. Two primary feeding groups of *O. mykiss*: CSNPs-treated and non-treated control. After 21 days feeding, all fish were immersed with *Y. ruckeri* (CSF007-82) for 2 h except for one negative, pathogen-free group subset. Fish were sampled at five specific time-points, including 0, and 21st day post feeding (dpf), and 5th, 10th, and 28th day post infection (dpi).

After acclimation, 180 apparently healthy *O. mykiss* were selected for the in vivo experiment (extended for 49 days). Twelve 75 L fiber glass indoor tanks with a primary stocking density of 15 fish per tank were assigned into two treatment groups for a feeding trial extended for 21 days, and then re-assigned into four subset test groups for a subsequent post-challenge trial extended for 28 more days (Scheme 2). The ‘tank effects’ were considered during the experiment, where the differences between tanks were lowered as possible and the fish of the replicate tanks were exposed to similar conditions [75,76]. For the feeding trial, fish were randomly and equally distributed into six replicate tanks per group and fed twice daily *ad libitum*. One group received medicated feed supplied with CSNPs and served as the CSNPs-treated group; while the other group received non-medicated commercial feed and served as the non-treated control group. For the post-challenge trial, each feeding group was split out into two subset groups giving rise to four experimental groups. The non-treated control group was split out into a negative (pathogen-free) and a positive (challenged) control groups, while the CSNPs-treated group was split out into a prophylactic-regimen (short-term treated) and a therapeutic-regimen (long-term treated) subset groups. All groups were challenged with *Y. ruckeri*, except for the negative pathogen-free control group. All fish received the same feed as before until the end of the experiment, except for the prophylactic-regimen group that was stopped receiving the medicated feed and started feeding with non-medicated feed. The post-challenge trial continued for 28 days, and then the fish were collected and processed for tissue analysis. Throughout both trials, the tanks were kept continuously clean via siphoning the leftover wastes and feed one hour after each feeding time.

### 4.7. Bacterial Challenge

ERM infection was performed using *Y. ruckeri* (CSF007-82) virulent isolate that was obtained from the National Centre for Cool and Cold Water Aquaculture, Kearneysville, West Virginia, USA, and stored at −80 °C in the Clinical Division of Fish Medicine, University of Veterinary Medicine, Vienna. Prior use, one colony was inoculated in tryptic soy (TS) broth (Sigma-Aldrich, Austria) and was cultured overnight at 22 °C in an orbital incubator, 144 revolutions per minute (rpm) [31]. For the experimental challenge, the bacterial suspension was adjusted at a theoretical concentration of 2 × 10^9^ CFU ml^−1^, which was assessed using a spectrophotometer (Eppendorf BioPhotometer^®^, Eppendorf, Hamburg, Germany). Water level was lowered to reach 50 L/tank, then the bacterial challenge was performed by 2 h bath exposure to the bacterial suspension to reach a final concentration of 2 × 10^6^ CFU ml^−1^ [77]. Pathogen-free group was exposed to sterile phosphate-buffered saline (PBS) (Sigma-Aldrich, Austria). For spreading the infection, the water flow was temporarily stopped while the airflow was constantly maintained. Then, the water flow was resumed after 2 h of exposure.

### 4.8. Fish Sampling and Tissue Collection

Three fish from each replicate tank were sampled on each one of five time-points including, zero, and 21st dpf to evaluate the impact of CSNPs feeding on fish health, and on the 5th, 10th, and 28th dpi for comparing the prophylactic and the therapeutic efficacy of dietary CSNPs against ERM infection. The fish were fasted for 24 h prior sampling to be dissected at the post-absorptive state [16]. Before euthanasia, skin mucus was collected via gentle head to tail dorsolateral scrapping using sterile spatula thereby avoiding bleeding or fecal contamination. After mucus scrapping, fish were euthanized using an overdose (300 mg L^−1^) of tricaine methane sulfonate (MS-222) and blood samples were collected from fish caudal veins using non-heparinized syringes. Mucus or blood of all the three fish sampled from each individual tank were collected together (i.e., one pool/tank) to provide sufficient amounts for analysis [40]. One fish from each tank was dissected and the entire intestine was excised and discharged from its fecal content via gentle squeezing along the exterior side by using a sterile scalpel. The emptied intestines were washed via rinsing in PBS and cut into small pieces. Anterior intestinal pieces, which are the main sites of nutrient absorption, were fixed in 10% neutral buffered formalin for examination of the histological or the histopathological traits. Mid-gut and posterior intestinal pieces were kept in RNAlater™ (Sigma-Aldrich, Austria) at 4 °C for total RNA extraction and gene expression analysis.

### 4.9. Analysis and Measurement

#### 4.9.1. *Oncorhynchus mykiss* Health after Three Weeks Feeding Trial

I. Growth Performance and Fish Survival

An individual enumeration along with body weight assessment of the sampled fish was assessed on zero time-point and the 21st dpf. The data were processed through assessing the survival and growth performance (weight gain and the specific growth rate) calculations according to Basuini et al. [78].

II. Intestinal Histological Appraisals

Formalin fixed anterior intestinal segments were processed for the normal histological procedures of paraffin blocks embedding, and ultrathin sectioning (4 μm) using Microm HM 360^®^microtome according to Suvarna et al. [79]. Usual H&E counter staining was conducted to investigate the histological traits after the short-term feeding trial with CSNPs. Triplicate cross-sections per group were examined under a light microscope (Olympus BX 46) and were digitally photographed using its connected Olympus DP 21 digital camera (Olympus Corporation, Tokyo, Japan). The digital images were examined for the length of five longest, intact villi in the lumen of each one of the triplicate post-feeding images. Villi length was measured according to Najafabad et al. [47].

#### 4.9.2. *Oncorhynchus mykiss* Resistance against ERM Infection

I. Fish Antibacterial Defense

The antibacterial activity of *O. mykiss* serum and skin mucus against the growth of the virulent isolate *Y. ruckeri* (CSF007-82) was estimated in vitro. The bacterial growth inhibition exhibited by the CSNPs-treated samples was compared with that demonstrated by the non-treated samples using the same challenge concentration (2 × 10^6^ CFU mL^−1^). The skin mucus defense activity was evaluated via the serial dilution and the standard agar discs diffusion assays, while the serum defense activity was evaluated using the serum bactericidal activity assay.

II. Skin Mucus Antibacterial Activity

The agar discs diffusion assay was performed for the estimation of the ZOI demonstrated by the effect of *O. mykiss* crude mucus following the methods described by Taei et al. [80] with minor modification. Crude mucus was prepared via centrifugation (5000 rpm, 5 min at 4 °C) of the raw mucus freshly scraped from the sampled fish skin [45]. After inoculation, TS broth (100 µL) was spread on each one of several plates containing 15 mL TS agar by using T-shaped sterile lope. Three discs (6 mm diameter) supplied with 100 µL crude mucus, sterile Tris buffered (TB) saline (50 mM Tris HCl, pH 8.0, 150 mM NaCl, Sigma-Aldrich, Austria) (negative control), or distilled water (blank control) were placed on each one of the triplicate agar plates per tank. The plates were incubated overnight at 22 °C, and the clear diagonal zones showing no visible bacterial growth around the discs were measured in millimetres using a ruler.

Serial dilution assay was performed for assessing the MIC of *O. mykiss* mucus filtrates according to the methods described by Saeidi asl et al. [16] with a slight modification. Mucus filtrates were prepared via vigorous shaking of equal volumes from the fresh mucus and sterilized TB saline followed by 15 min centrifugation (30,000 g, 4 °C) and supernatants filtration on Whatman no.1 filter papers. Different amounts (50, 100, 150, and 200 μL) from the mucus filtrate from fish of each tank were placed inside serial subsequent triplicate tubes, each containing 1 mL inoculated TS broth. Sterile TS broth replaced the inoculated broth in the blank control tubes, while sterile TB saline replaced the mucus in the negative control tubes. The tubes were incubated overnight at 22 °C, and the lowest concentration showed no visible growth was considered the MIC value.

III. Serum Bactericidal Activity

Initially, sera were prepared via centrifugation (3000g, 15 min, at 4 °C) [78] of the clotted blood that were kept for 4 h at 4 °C. Subsequently, serum bactericidal activity was assessed following the methods described by Guardiola et al. [40] with some modifications. Each one of serial dilutions (1/10) from the bacterial suspension was mixed with equal volume (50 µL) from sera in different triplicate wells of a 96-well microtiter plate. Sterile TS broth replaced the inoculated broth in the blank control wells, while PB saline replaced the serum in the positive control wells. The plates were incubated for 24 h at 22 °C, then shaken slightly in an orbital incubator for 10 min [30]. The optical density (OD) for each well was measured at 620 nm and the blank control wells corresponding to each group were specified as blanks for the OD measurements. The bacterial growth inhibition was calculated for each experimental group according to Ahmed et al. [31].

Inhibition percent (%) = [1 − ((OD sample)/(OD positive control))] × 100.(1)

#### 4.9.3. *Oncorhynchus mykiss* Intestinal Immunity Investigations

I. Histochemical Examination

Histochemical characterization and differentiation of the intestinal mucous (goblet) cells was performed on the paraffin sections of the *O. mykiss* lower intestine sampled at all the time-points included in both of the experimental trials. It was conducted in accordance with Padra et al. [81] by employing AB&PAS double staining for their color differentiation according to their mucin content. AB (pH 2.5) stains the acid mucin-producing cells blue, while PAS stains the neutral mucin-producing cells pink. Triplicate cross-sections per each group were examined under the light microscope and were digitally photographed using its connected camera. The digital images were investigated for the semi-quantification of the goblet cells based on their gained colors as proposed by Jung-Schroers et al. [56], but along 1000 µm epithelial length at three different fields per slide. Cells, which are negatively stained (free of mucin), and which are differentially stained blue (producing acid mucin), pink (producing neutral mucin), or purple (producing mixed acid/neutral mucin) were differentiated and counted individually along the specified areas. The counting was assessed as blinded samples to exclude the biased evaluation. Data were expressed as mean percent ± SD.

II. Gene-Transcription Analysis

Total RNA extraction and relative mRNA expression analysis were conducted mainly to study the expression pattern of four intestinal immune-related genes (IL-1β, TGF-β, LYZ II, and IgM H) after dietary medicated or non-medicated diets, and in response to ERM infection. Elongation factor-alpha (EF1-α) and β-actin were the housekeeping genes for normalizing the expression of our target genes through the reverse transcriptase quantitative polymerase chain reaction (RT-qPCR) assay [82]. The analysis was carried out on a CFX96 Touch Real-Time PCR detection system (Bio-Rad, Munich, Germany) with qPCR SYBR Green Supermix and the gene-specific primers. The sequences, accession numbers, and the efficiency of the primers used in the current analysis are listed in Table 4.

RNeasy Mini Kit (Qiagen, Hilden, Germany) was used for the total RNA extraction with including an on-column DNase I digestion step (Qiagen, Hilden, Germany). The extracted RNA was qualified and quantified on a NanoDrop 2000 spectrophotometer (Thermo Fisher Scientific, Vienna, Austria), and the cDNA was synthesized from 1 µg mRNA input according to the user’s manual by using of iScript cDNA synthesis kit (Bio-Rad, Hercules, Munich, Germany) [85]. Duplicate template and transcriptase controls were included to assess the performance of the qRT-PCR assay for each gene. The qPCR analysis was performed on 10 µL final volume (5 µL of 5X Sso Advanced™ Universal SYBR Green Supermix (Bio-Rad), 3 µL DNA-free water (Bio-Rad, Hercules, USA), 0.5 µL of each primer (5 pmol/µL), and 1 µL of 1:5-fold diluted cDNA). Gene expression cycling was setup in four subsequent steps including, an initial step for cDNA initial denaturation (5 min at 95 °C), final denaturation step (45 cycles, 15 s at 95 °C), primer annealing step (15 s at 55 °C), and a final step for extension of the double stranded cDNA (72 °C for 15 s). Melting curve analysis for detection the non-specific primers binding was performed at the end of each cycling starting from 55 °C up to 95 °C via gradual increase of 0.5 °C every 10 s. The fold change in mRNA relative expression between the experimental groups at each sampling time-point was determined based on 2^−∆∆Ct^ method, and CFX Maestro Software (Bio-Rad Laboratories Inc., Munich, Germany) was used for assessing the expressions normalization.

#### 4.9.4. Disease Progression

I. Fish Mortality

Fish were closely monitored three times a day over 28 days after the bacterial challenge for following up the disease progression and fish resistance after challenge. The clinical signs associated with disease progression were recorded to evaluate the differences in fish resistance between the experimental groups. Moribund fish were euthanized humanely by MS-222 overdose to avoid their suffering. The moribund fish of each challenged group were recorded as its mortality count for cumulative mortality assessment. The RPS was calculated in relative to the challenged non-treated control group by the following equation according to Yarahmadi et al. [86].

RPS (%) = [1 − ((Group mortality (%))/(Challenged non-treated control group mortality (%)))] × 100(2)

II. Histopathological Examination

The histopathological traits associated with the ERM infection were examined on the H&E counter-stained cross-sections of the *Y. ruckeri* challenge samples.

### 4.10. Statistical Analysis

The statistical analysis was conducted on the normally distributed data using the statistical package for social sciences (SPSS) version 25 (IBM Corporation, Armonk, NY, USA) software. Data were obtained from ≥ 3 independent replicates and expressed as means ± SD. Comparison between groups was conducted via covariance analysis (ANOVA), and the differences between them were tested for the statistical significance with Duncan post hoc test for the paired comparison of means [87]. Differences between data sets were considered statistically significant at *p* < 0.05 and > 95% confidence level. *p*-values of < 0.01 and < 0.001 were considered highly significant and very highly significant, respectively.

## 5. Conclusions

The present study showed that the marine bio-sourced CSNPs were effective as immunomodulatory feed additive for *O. mykiss* resistance against ERM infection via enhancing its antimicrobial defense ability and intestinal immunity. Our findings refer positively to the impact of supplying fish diets with CSNPs for the activation of their intestinal immune-relevant gene expression for higher intestinal inflammatory responses against infections. Additionally, our findings give an insight about its role in the enhancement of fish antibacterial defense for controlling pathogens invading. However, the long-term therapeutic-regimen administration might be more appropriate than the prophylactic-regimen administration since it provides higher gut protection and disease resistance.

## Figures and Tables

**Table 1:** Growth performance and survival of *O. mykiss* fed with CSNPs (5 g Kg^−1^ dry feed weight) for 21 days.

Treatment Group	Parameters				
	IBW (gm)	FBW (gm)	WG (%)	SGR (%)	Survival (%)
Non-supplemented	15.36 ± 0.69 ^a^	26.36 ± 0.39 ^a^	71.86 ± 6.60 ^a^	2.58 ± 0.18 ^a^	100 ^a^
CSNPs-treated	15.70 ± 0.62 ^a^	27.12 ± 0.59 ^b^	72.99 ± 7.11 ^a^	2.61 ± 0.19 ^a^	100 ^a^

**Table 2:** Bacterial growth inhibition zones (ZOI, mm), and the minimum inhibitory concentrations (MIC µL mL^−1^) displayed against the virulent isolate *Y. ruckeri* (CSF007-82) by the skin mucus of *O. mykiss*, fed on non-supplemented diets or diets supplemented with CSNPs (5g kg^−1^ dry feed weight) and sampled at day zero and 21st day post feeding (dpf).

Treatment Group	Parameters/Sampling Day			
	ZOI		MIC	
	Day zero	21st dpf	Day zero	21st dpf
Non-supplemented	2.34 ± 0.12 ^a^	2.54 ± 0.16 ^a^	>200	>200
CSNPs-treated	2.86 ± 0.19 ^a^	4.28 ± 0.67 ^b^	>200	150

**Table 3:** Bacterial growth inhibition zones (ZOI, mm), and the minimum inhibitory concentrations (MIC, µL mL^−1^) displayed against the virulent isolate *Y. ruckeri* (CSF007-82) throughout the post-challenge trial by the skin mucus of *O. mykiss*, fed on non-supplemented diets or fed on diets supplemented with CSNPs (5g kg^−1^ dry feed weight) by two different regimens.

Treatment	Subset Group	Parameters/Day Post Infection(dpi)					
		ZOI			MIC		
		5th dpi	10th dpi	28th dpi	5th dpi	10th dpi	28th dpi
Non-supplemented	Pathogen-free Control	2.66 ± 0.24 ^a^	2.52 ± 0.19 ^a^	2.67 ± 0.21 ^a^	>200	>200	>200
	Challenged Control	5.07 ± 0.38 ^b^	3.45 ± 0.25 ^b^	2.63 ± 0.16 ^a^	150	200	>200
CSNPs-treated	Prophylactic-regimen	5.26 ± 0.88 ^b^	3.53 ± 0.22 ^b^	2.72 ± 0.17 ^a^	100	200	200
	Therapeutic-regimen	7.50 ± 0.26 ^c^	7.16 ± 0.21 ^c^	8.08 ± 0.34 ^b^	100	100	50

**Table 4:** List of primers used for the qRT-PCR gene expression analysis

Primer (Gene) Name	Primer Sequence (5′-3′)	Gene Bank Accession No.	Amplicon Size (bp)	Reference
β-actin	F: ATGGAAGGTGAAATCGCC R: TGCCAGATCTTCTCCATG	AF157514	260	[1]
EF1-α	F: AGACAGCAAAAACGACCCCC R: AACGACGGTCGATCTTCTCC	HF563594	167	[83]
IL1-β	F: TATCCCATCACCCCATCACC R: GGGCGTGACGTACGAAGACAGG	AJ223954	357	This study
LYZ II	F: GCTGTTGTTCTCCTGCT R: GCAAACCCAGTTGGGCAG	NM_001124716.1	129	[84]
IgM H	F: ACTGCTCCGACTTTGTTCCC R: CCGCAGGGTACTGAACGAAA	XM_036941450.1	160	[83]
TGF-β	F: AGTTGCCTTGTGATTGTGGG R: GGGGTGGGCAGAGGCTCCGG	X99303	404	This study

## Data Availability

Data is contained within the article or supplementary material.

## Supplementary Materials

The following are available online at https://www.mdpi.com/1660-3397/19/2/72/s1, Figure S1: Size characterization of CSNPs prepared in the current study. (a) Particle size by intensity peak referring at 100 nm; (b) Particle size distribution curve showing narrow size distribution range from 73 to 145 nm (red column chart), and homogenous distribution of the particles (green disciplined Z-shaped peak). The data were expressed as means ± SD (n = 3); Figure S2: Microscopic characterization of CSNPs synthesized in the current study. (a) SEM micrograph showing homogenous spherical particles without agglomerations (scale bar = 0.5 μm); (b) TEM micrograph showing regular spherical-shaped particles (scale bar = 250 nm); Figure S3: X-ray diffraction pattern of (a) chitosan; (b) CSNPs.
