# Supplementary material for: Dietary Chitosan Nanoparticles: Potential Role in Modulation of Rainbow Trout (Oncorhynchus mykiss) Antibacterial Defense and Intestinal Immunity against Enteric Redmouth Disease
Source: Mar Drugs. 2021 Jan 29;19(2):72. doi: 10.3390/md19020072 (PMC7911277; doi:10.3390/md19020072)
Supplement: Supplementary file 1 [file marinedrugs-19-00072-s001.pdf]

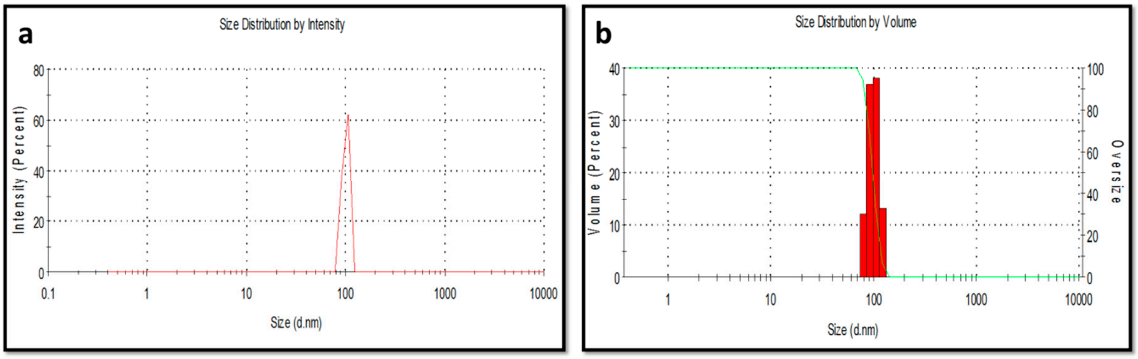

Figure S1. Size characterization of CSNPs prepared in the current study. (a) Particle size by intensity peak referring at 100 nm; (b) Particle size distribution curve showing narrow size distribution range from 73 to 145 nm (red column chart), and homogenous distribution of the particles (green disciplined Z-shaped peak). The data were expressed as means ± SD (n = 3).

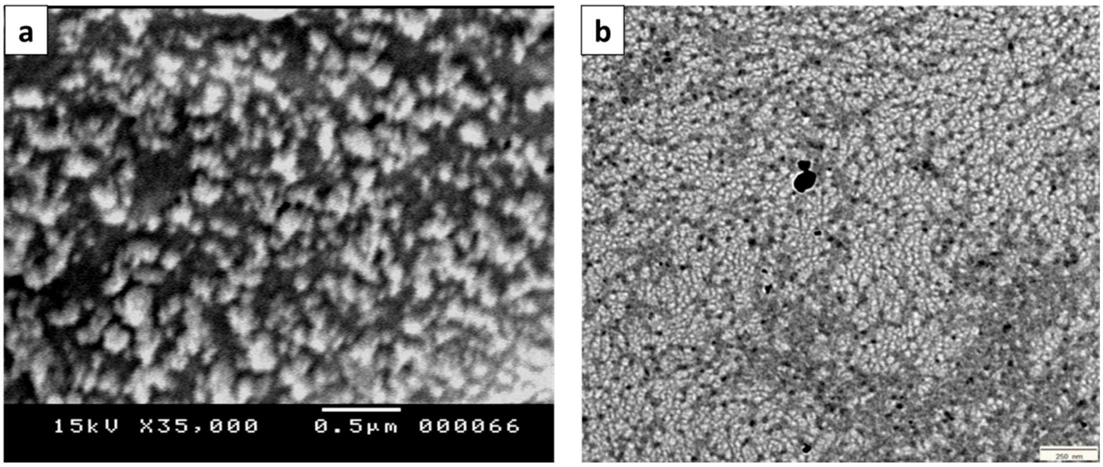

Supplementary 2. Microscopic characterization of CSNPs synthesized in the current study. (a) SEM micrograph showing homogenous spherical particles without agglomerations (scale bar = 0.5 μm); (b) TEM micrograph showing regular spherical-shaped particles (scale bar = 250 nm).

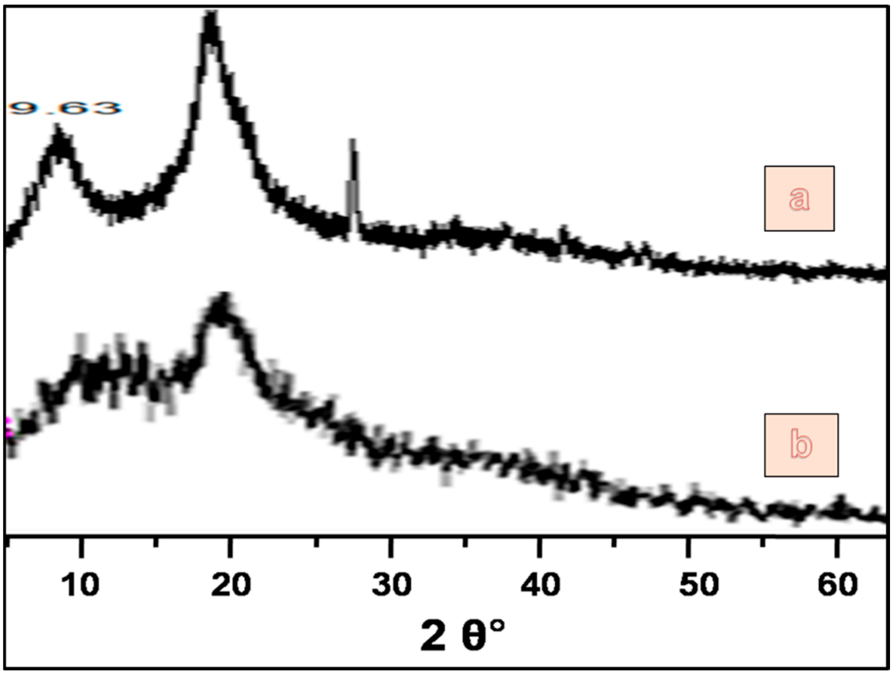

Supplementary 3: X-ray diffraction pattern of (a) chitosan; (b) CSNPs.
